# Cytokine and chemokine responses to helminth and protozoan parasites and to fungus and mite allergens in neonates, children, adults, and the elderly

**DOI:** 10.1186/1742-4933-10-29

**Published:** 2013-07-15

**Authors:** Christian J Lechner, Karl Komander, Jana Hegewald, Xiangsheng Huang, Richard G Gantin, Peter T Soboslay, Abram Agossou, Meba Banla, Carsten Köhler

**Affiliations:** 1 Institute for Tropical Medicine, Eberhard Karls Universität Tübingen, Wilhelmstrasse 27, Tübingen 72074, Germany; 2 Onchocerciasis Reference Laboratory, Institut National d’Hygiène, Sokodé, Togo; 3 Centre Hospitalier Régional, Service Pédiatrie, Sokodé, Togo; 4 Centre Hospitalier Universitaire Campus de Lomé, Université de Lomé, Lomé, Togo

**Keywords:** Helminth, Protozoa, Parasite infection, Cytokine, Chemokine, Cellular response, Age groups

## Abstract

**Background:**

In rural sub-Saharan Africa, endemic populations are often infected concurrently with several intestinal and intravascular helminth and protozoan parasites. A specific, balanced and, to an extent, protective immunity will develop over time in response to repeated parasite encounters, with immune responses initially being poorly adapted and non-protective. The cellular production of pro-inflammatory and regulatory cytokines and chemokines in response to helminth, protozoan antigens and ubiquitous allergens were studied in neonates, children, adults and the elderly.

**Results:**

In children schistosomiasis prevailed (33%) while hookworm and *Entamoeba histolytica/E. dispar* was found in up to half of adults and the elderly. *Mansonella perstans* filariasis was only present in adults (24%) and the elderly (25%). Two or more parasite infections were diagnosed in 41% of children, while such polyparasitism was present in 34% and 38% of adults and the elderly. Cytokine and chemokine production was distinctively inducible by parasite antigens; pro-inflammatory Th2-type cytokine IL-19 was activated by *Entamoeba* and *Ascaris* antigens, being low in neonates and children while IL-19 production enhanced “stepwise” in adults and elderly. In contrast, highest production of MIP-1delta/CCL15 was present in neonates and children and inducible by *Entamoeba-*specific antigens only. Adults and the elderly had enhanced regulatory IL-27 cytokine responses, with Th2-type chemokines (MCP-4/CCL13, Eotaxin-2/CCL24) and cytokines (IL-33) being notably inducible by helminth- and *Entamoeba-*specific antigens and fungus-derived allergens. The lower cellular responsiveness in neonates and children highlighted the development of a parasite-specific cellular response profile in response to repeated episodes of exposure and re-infection.

**Conclusions:**

Following repeated exposure to parasites, and as a consequence of host inability to prevent or eliminate intestinal helminth or protozoa infections, a repertoire of immune responses will evolve with lessened pro-inflammatory and pronounced regulatory cytokines and chemokines; this is required for partial parasite control as well as for preventing inadequate and excessive host tissue and organ damage.

## Background

Parasitic infections are common in countries with poor hygienic conditions, where a lack of sanitation and health care facilitates the transmission and the spread of helminths like *Ascaris lumbricoides, Schistosoma spp.*, hookworms, and protozoa like *Entamoeba histolytica/dispar*[1]. Approximately two billion people are infected with helminth parasites worldwide, and some intestinal parasites may affect up to 70% of an endemic population [2]. The dispersion of parasites often overlaps, and individuals living in such areas acquire multiple infections during their lifetime and are infected concurrently with several parasite species [1]. The encounter with parasite species elicits distinct and specific immune responses in their host; cytokines and chemokines are key players which regulate and polarize cellular reactivity and antibody responses to antigens and allergens. Helminth infections associate with an initial Th1 immune response during pre-patency, while during patency a Th2-type response prevails [3,4]. Generally with chronic helminth parasite infections Th2-type cytokine responses predominate [5], while Th1-type cytokine responses are important for protection against protozoa, e.g. amoebiasis [6] or *Plasmodium falciparum* malaria [7].

A specific, balanced and, to an extent, protective immunity develops over time in response to repeated parasite encounter, with immune responses initially being poorly adapted and non-protective [8,9]. Such incapability can result in parasite persistence and host tissue damage as a result of inappropriate inflammatory reactivity. With repeated episodes of infection, parasite clearance, and re-infection, immune responses to foreign antigens become increasingly specific and effective; however, the development of such immunity with increasing age is not well understood. Cytokines and chemokines may enhance, suppress, and regulate the expression of immunity to intravascular and intestinal parasites; moreover, they particularly promote chemotaxis and the activation of effecter cells in parasite-invaded tissues and cells.

Monocyte chemoattractive proteins such as MCP-4 recruit effecter cells [10] and inflammatory proteins such as MIP-1delta/CCL15 and Eotaxin2/CCL24 activate eosinophil granulocytes, monocytes, and lymphocytes and so contribute to inflammation [11-13]. Cytokines released as “alarmins” and mediators of inflammation, examples being IL-19 and IL-33, may enhance Th2 type immune reactions during infections with intestinal nematodes [14-17], while IL-19 promotes chemotaxis of neutrophil granulocytes and the production of IL-6 and TNF-alpha [18,19]. Regulatory cytokines such as IL-27 limit exacerbating Th17 and Th2 responses [20] and decrease immune pathology during malaria infection [21,22]. While with several cytokines and chemokines their role in parasitic infections has not yet been investigated, others like Eotaxins were found to be important for effecter cell recruitment during helminth infection.

In order to further clarify the extent to which these immune mediators become activated or suppressed during early life parasite exposure, and also with a view to later life chronic pathogen persistence, we studied the cellular production of pro-inflammatory and regulatory cytokines and chemokines in neonates, children, adults, and the elderly in response to helminth and protozoa infectious challenge and to ubiquitous allergen exposure. Distinctive production levels were observed between these groups, highlighting the development of a parasite-specific cellular responsiveness to repeated episodes of exposure and re-infection.

## Results

### Helminth and protozoa parasite infections in children, adults, and the elderly

The prevalence of parasite infections and parasite co-infections in children, adults, and the elderly is shown in Table 1. Free of parasite infection were 37% of the children, while 29% of adults and 21% of the elderly were negative for parasites in urine, blood, and stools. In children schistosomiasis prevailed (33%) while in adults and the elderly *Schistosoma mansoni* or *S. haematobium* were less than 10%. Hookworm infections were present in 22%, 26% and 34% of children, adults and the elderly, respectively, while *E. histolytica/dispar* was found in 22%, 37% and 55% of same. *M. perstans* filariasis was present only in adults (24%) and the elderly (25%). Multiple parasite infections were diagnosed in 41% of children, while such polyparasitism was present in 34% and 38% of adults and the elderly.

**Table 1:** Prevalence of parasite infections and parasite co-infections in children, adults and elderly

	**Children**	**Adults**	**Elderly**
	**(n=35)**	**(n=39)**	**(n=42)**
*Schistosoma haematobium/S. mansoni*	33%	8%	2%
Hookworm	22%	26%	34%
*Entamoeba histolytica/E. dispar*	22%	37%	55%
*Mansonella perstans*	0%	24%	25%
Infection-free	37%	29%	21%
Single parasite infection	22%	37%	41%
Multiple parasite infections	41%	34%	38%

Fresh stool samples were analyzed by microscopy for intestinal helminth eggs as well as protozoan cysts and trophozoites, and all stool samples were examined using the Kato-Katz technique. *Schistosoma haematobium* eggs were detected by filtration of urine samples and subsequent microscopic examination of filters, while microfilaria of *Mansonella perstans* were detected by microscopic analysis after gradient density centrifugation of whole blood samples as detailed in Materials and Methods.

### Cellular cytokine responses to helminth and protozoa parasite antigens in neonates, children, adults, and the elderly

Table 2 shows the antigen-inducible production levels of IL-19, IL-27, and IL-33 by umbilical cord blood cells (UCBC) and peripheral blood mononuclear cells (PBMC) (Data not shown for Asc and Ov). In neonates and children IL-19 did not change between not stimulated (Base) and antigen-stimulated UCBC and PBMC. In adults IL-19 production enhanced following *Ascaris* (Asc) and *Entamoeba* (Eh) antigens stimulation (for Eh p<0.05 compared to Base). In the elderly, Eh, *O. volvulus* (Ov) and Asc antigens stimulated IL-19 responses (for Eh p<0.05 compared to Base). The production levels of IL-27 were low in neonates and children, but IL-27 production enhanced overall in adults and the elderly, without significant differences between the groups. Spontaneous cellular release of IL-33 as well as amounts produced in response to antigens from Eh, Ov and *Plasmodium* (Pf) remained low in all groups; only in response to Asc antigen were UCBC and PBMC from all four study groups found to secrete elevated amounts of IL-33 (p<0.01 for children and adults, p<0.001 for the elderly; compared to Base).

**Table 2:** Cytokines and Chemokines in response to parasite antigens

**Cytokine/chemokine**	**Study group**	**Base**	**Eh**	**Pf**
IL-19	Neonates	137 (0/301)	240 (77/404)	271 (9/449)
	Children	204 (81/327)	382 (229/535)	189 (36/341)
	Adults	244 (114/375)	692 * (507/877)	370 (186/555)
	Elderly	257 (128/386)	726 * (544/909)	319 (137/502)
IL-27	Neonates	425 (307/542)	495 (378/612)	518 (393/642)
	Children	557 (429/686)	666 (513/820)	501 (340/661)
	Adults	719 (503/935)	1004 (699/1309)	886 (581/1191)
	Elderly	1050 (898/1202)	951 (738/1164)	932 (737/1128)
IL-33	Neonates	52 (17/86)	67 (31/104)	32 (0/71)
	Children	22 (0/110)	107 (2/212)	14 (0/123)
	Adults	29 (5/54)	51 (16/87)	52 (28/77)
	Elderly	45 (28/62)	76 (26/125)	62 (36/88)
MCP-4/ CCL13	Neonates	69 (40/99)	56 (26/86)	51 (19/82)
	Children	180 (120/240)	54 (0/126)	85 (40/130)
	Adults	129 (84/175)	14 (8/20)	135 (88/183)
	Elderly	124 (85/163)	12 (0/67)	133 (78/188)
Eotaxin-2/ CCL24	Neonates	537 (337/738)	540 (340/741)	419 (205/632)
	Children	358 (264/454)	319 (201/438)	360 (235/486)
	Adults	1398 (1223/1572)	544 **(297/790)	1762 (1515/2008)
	Elderly	1430 (1262/1598)	340 *** (102/577)	1405 (1168/1643)
MIP-1delta/ CCL15	Neonates	63 (47/79)	68 (52/83)	64 (47/80)
	Children	46 (41/62)	56 (43/68)	41 (28/55)
	Adults	19 (12/29)	35 (24/46)	24 (13/34)
	Elderly	27 (16/37)	63 ** (49/78)	26 (12/41)

The cellular production of interleukin (IL)-19, IL-27, IL-33, MCP-4/CC13, MIP-1delta/CCL15 and Eotaxin-2/CCL24 in response to parasite antigens by umbilical cord blood cells (UCBC) and peripheral blood mononuclear cells (PBMC) from different age groups is shown. UCBC from neonates (n=36) and PBMC were isolated from children (age 10-13 years; n=35), adults (18-45 years, n=39) and the elderly (50-80 years, n=42), and stimulated *E. histolytica* antigen (Eh; 10 μg/ml), *P. falciparum* antigen (Pf; 1x10^8^ schizonts/ml) or not stimulated (Base), for 48 hours at 37%, 5% CO_2_ and saturated humidity. Cytokine and chemokine concentrations in cell culture supernatant were determined by specific enzyme-linked immunosorbent assays (ELISA). The data are given as means with the lower and upper 95% confidence intervals (in brackets). (* p<0.05, ** p<0.01 ***p<0.001, compared to Base).

### Cellular chemokine release to helminth and protozoan parasite antigens in neonates, children, adults, and the elderly

Cellular production of MCP-4/CCL13, MIP-1delta/CCL15 and Eotaxin-2/CCL24 by UCBC and PBMC following stimulation with helminth- and protozoa-specific antigens is shown in Table 2 (Data not shown for Asc and Ov). Parasite antigen-induced MCP-4/CCL13 production by UCBC from neonates did not differ from spontaneous release. In children, baseline MCP-4/CCL13 production did not enhance when their PBMC were cocultured with Eh, Ov and Pf antigens. Similarly, in adults and the elderly, Eh and Pf antigens did not heighten MCP-4/CCL13 production, while Asc- and Ov-specific antigens strongly activated MCP-4/CCL13 by PBMC (p<0.01, when compared to Base).

Production of the pro-inflammatory Eotaxin-2/CCL24 was low in neonates and children, irrespective of the antigens used for cell stimulation. In adults, *Ascaris* and also *Onchocerca* and *Plasmodium* antigen extracts enhanced Eotaxin-2/CCL24 release (p<0.01 for *Ascaris* when compared to Base), while stimulation with *Entamoeba* antigen significantly reduced Eotaxin-2/CCL24 levels (p<0.01 compared to Base). In the elderly, Eotaxin-2/CCL24 production enhanced in response to *Ascaris* (p<0.05), while depressed in response to *Entamoeba* antigen (p<0.001). Highest levels of MIP-1delta/CCL15 were produced by UCBC and PBMC from neonates and children, with no significant differences between the individual antigen stimulations. Overall MIP-1delta/CCL15 production was lower in adults (mean children 62 pg/ml, mean adults 25 pg/ml); only *Entamoeba* antigens slightly increased MIP-1delta/CCL15 production, with this *Entamoeba* antigen-specific activation being significant in PBMC from the elderly (p<0.01). All other parasite antigens elicited MIP-1delta/CCL15 production levels around baseline levels (Table 2).

### Helminth antigen-induced cellular production of cytokines (IL-19, IL-27, IL-33) and chemokines (MCP-4/CCL13, MIP-1delta/CCL15, Eotaxin-2/CCL24)

**Figure 1:**
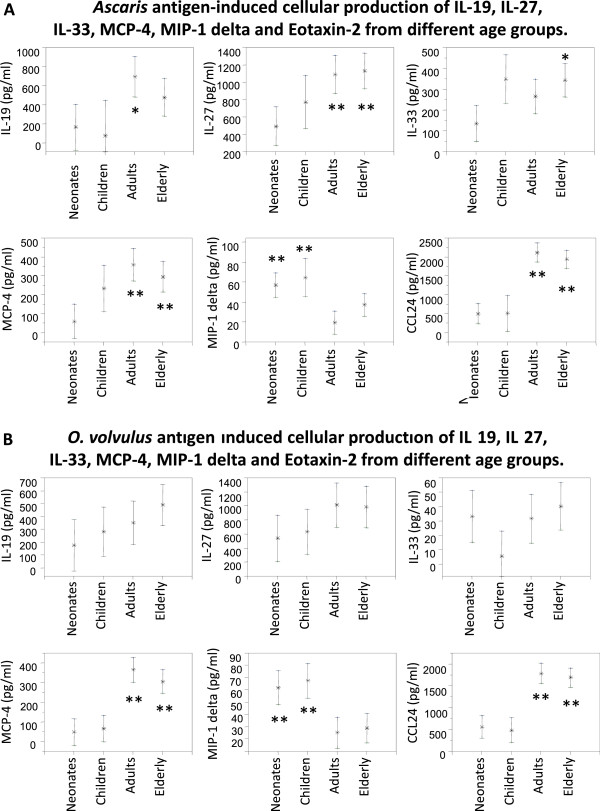
**Cellular production of interleukin (IL)-19, IL-27, IL-33 MCP-4/CCL13, MIP-1delta/CCL15 and Eotaxin-2/CCL24 in response to *A. lumbricoides *(Asc) and *O. volvulus *(Ov) antigen by umbilical cord blood cells (UCBC) and peripheral blood mononuclear cells (PBMC) from different age groups is shown. A**. Ascaris antigen-induced cellular production of IL-19, IL-27, IL-33, MCP-4, MIP-1delta and Eotaxin-2 from different age groups. **B**. O. volvulus antigen-induced cellular production of IL-19, IL-27, IL-33, MCP-4, MIP-1delta and Eotaxin-2 from different age groups.

The responses of UCBC and PBMC from neonates, children, adults, and the elderly to helminth-specific *A. lumbricoides* (Figure 1A) and *O. volvulus* antigens were evaluated (Figure 1B). Cellular response of IL-19, IL-27, IL-33 and MCP-4/CCL13 to *Ascaris* antigen was lowest in neonates, and enhanced in children, adults, and the elderly (Figure 1A). Highest production levels of IL-19, IL-27 and MCP-4/CCL13 were observed in adults and the elderly, without differences between the age-groups (Figure 1A). Eotaxin-2/CCL24 production was highly elevated in adults and the elderly as compared to neonates and children (p<0.01). In contrast, Asc antigen-induced production of MIP-1delta/CCL15 was clearly lower in adults than in neonates and children (p<0.01 compared to neonates). Broad confidence intervals for IL-19 and IL-33 were observed in neonates and children for all cytokines, while confidence intervals were smaller in adults (Figure 1A).

*O. volvulus* antigen-specific production of IL-19 while low in neonates was found to be enhanced “stepwise” in children, adults, and the elderly (Figure 1B). UCBC and PBMC from neonates and children produced lower amounts of IL-27 than did PBMC from adults and the elderly. Mean levels of IL-33 as produced by UCBC and PBMC from neonates, adults, and the elderly were similar; only IL-33 production was decreased in children in response to Ov antigen. Production of MCP-4/CCL13 and Eotaxin-2/CCL24 was lowest in neonates and children, while PBMC from adults and the elderly produced significantly enhanced amounts of both chemokines (p<0.01, compared to neonates and children). In contrast, the production levels of MIP-1delta/CCL15 were highest in neonates and children, and significantly lower in adults and the elderly (p<0.01, compared to neonates and children) (Figure 1B).

### Fungus and mite allergen-induced cellular production of cytokines and chemokines in neonates, children, adults, and the elderly

Tables 3 and 4 shows the cellular production of cytokines (IL-19, IL-27, IL-33) and chemokines (MCP-4/CCL13, Eotaxin-2/CCL24, MIP-1delta/CCL15) by neonatal UCBC and PBMC in response to *A. fumigatus*, *D. farinae* and *D. pteronyssinus* extracts.

**Table 3:** **Cytokines and chemokines in response to*Aspergillus fumigatus*allergen extract**

	**IL-19**	**IL-27**	**IL-33**	**MCP-4**	**CCL-24**	**MIP-1 delta**
Neonates	49 (0/603)	311 (0/1120)	62 (0/148)	0 (0/162)	283 (0/1198)	42 (0/87)
Children	ND	1096 (957/1236)	97** (80/113)	ND	ND	ND
Adults	280 (151/411)	823 (589/1056)	34 (7/60)	116 (72/162)	1728 (1474/1982)	29 (17/41)
Elderly	297 (172/424)	1136 (911/1363)	56 (32/81)	119 (75/163)	1558 (1315/1802)	31 (19/43)

** p<0.01 in comparison to adults for IL-33.

Cellular production of interleukin (IL)-19, IL-27, IL-33 MCP-4/CCL13, MIP-1delta/CCL15 and Eotaxin-2/CCL24 in response to allergen extracts by umbilical cord blood cells from neonates (UCBC) or peripheral blood mononuclear cells (PBMC) from children (age 10-13 years), adults (18-45 years) and the elderly (50-80 years). UCBC or PBMC were stimulated with extracts from *A. fumigatus* (final concentration 20 μg/ml) (Part A) or *D. farinae* (20 μg/ml) and *D. pteronyssinus* (20 ug/ml) (Part B) for 48 hours at 37%, 5% CO_2_ and saturated humidity. Cytokine and chemokine concentrations in cell culture supernatant were determined by enzyme linked immunosorbent assay (ELISA). The means of the brut production and the lower and upper 95% confidence intervals (in brackets) are shown. *ND* Not determined.

**Table 4:** **Cytokines and chemokines in response to*Dermatophagoides farinae/pteronyssinus*allergen extracts**

	**IL-19**	**IL-27**	**IL-33**	**MCP-4**	**CCL-24**	**MIP-1delta**
Neonates	149 (39/260)	496 (330/662)	34 (14/55)	53 (28/79)	655 (448/862)	82(70/94)
Children	ND	1101*** (993/1208)	101*** (88/115)	ND	ND	ND
Adults	421 ** (328/514)	799 (640/958)	36 (17/56)	83 (59/107)	1249 (1059/1440)	26 (14/37)
Elderly	411 ** (321/501)	1077 (937/1217)	36 (20/51)	101 (78/124)	1377 (1194/1561)	25 (15/36)

For IL-19 and IL-27: ** p<0.01 in comparison to neonates, *** p<0.001 in comparison to neonates; for IL-33: *** p<0.001 in comparison to all groups.

Allergen-stimulated production of IL-19 and IL-27 was lowest in UCBC from neonates, while IL-19 production levels were found to be enhanced in adults and the elderly (for mite allergens p<0.01, compared to neonates). Cellular production of mite allergen-induced IL-27 was lowest in neonates but highly elevated in children. Both fungus (Af) and mite (Df) allergen induced cellular production of IL-33 were strongly elevated in children (p<0.01 and p<0.001), compared to neonates, adults, and the elderly). Allergen stimulation did not induce cellular MCP-4/CCL13, Eotaxin-2/CCL24 or MIP-1 delta/CCL15 production above baseline levels.

## Discussion

Immune memory and cellular effector responses against parasites may develop and take shape gradually with repeated exposure, pathogen persistence or their clearance, and also with re-infection. In the present work, cellular responsiveness in neonates and children was low while adults and the elderly had enhanced regulatory IL-27 cytokine responses, with Th2-type chemokines (MCP-4/CCL13, Eotaxin-2/CCL24) and cytokines (IL-19, IL-33) being inducible by parasite-specific antigens and allergens.

The pro-inflammatory Th2-type cytokine IL-19 while low in neonates and children was enhanced “stepwise” in adults and the elderly; IL-19 was predominantly activated by *Entamoeba* and *Ascaris* antigens and allergens also, signifying repeated encounter with protozoan and helminth parasites and environmental allergens – such responsiveness was not yet developed in neonates and children. High IL-19 levels were observed in patients with asthma and also in an allergen-inducible asthma animal model [18]. IL-19 was found to enhance IL-1beta, IL-6, and CXCL8/IL-8 release and to attract granulocytes [18,19]; such mobilized and activated effecter cells may then adhere and attack tissue-infiltrating and migrating helminth larvae of *Ascaris* and hookworm. Furthermore, antigens of *Ascaris suis* and *E. histolytica* elicited strong chemotaxis and production of superoxide anions in neutrophil granulocytes [23,24]. IL-19 is a member of the IL-10 family; secreted by monocytes, epithelial cells and B cells [25-27], it exerts regulatory effects and, in mice, protects against colonic inflammation [28] and induces Th2 responses [29]. Inducible cellular IL-19 production in adults and elderly could therefore mirror an adaptation to intestinal protozoan and metazoan parasite challenge and allergen exposure over time. Similarly to IL-19, low amounts of IL-33 were detected in neonates; only *Ascaris* antigen activated IL-33 in children, suggesting early life priming by intestinal helminths. As a member of the IL-1 family, IL-33 promotes the generation of Th2 immune responses by inducing secretion of IL-4, IL-5 and IL-13 by T cells [30]. High levels of IL-33 were detected in patients with asthma or allergic rhinitis [31,32]. The “Alarmin” IL-33 is released by injured epithelia and endothelia following hookworm infection so as to attract leukocytes to the site of inflammation [33,34]; moreover, IL-33 supports reduction and expulsion of the intestinal helminths *Heligmosomoides pylorus*, *Trichuris muris* or *Nippostrongylus brasiliensis* from infected mice [17,35,36]. A recent study [14] has disclosed the importance of IL-33 during murine hookworm infection: in IL-33 gene knockout mice infected with *N. brasiliensis*, cellular production of the Th2-type cytokine IL-13 was lessened and eosinophil recruitment reduced, and accompanied by a delayed worm expulsion in these animals.In the present work, allergens of mite and fungus activated IL-33 in children, suggesting that such increase in IL-33 reflects an initial responsiveness which, in later life and after repeated parasite exposure, is attenuated by regulatory cytokines like IL-27.

IL-27 production was low in neonates and children but gradually enhanced in adults and the elderly, with no difference between baseline and the antigen stimulation (Table 2). Mite allergen-induced production levels of IL-27 were highest in children, whereas in neonates, adults and the elderly, allergen stimulation did not induce IL-27 production above baseline levels (Tables 3 and 4). A member of the IL-12 family, IL-27 has been found to act as initiator and attenuator of immune responses [37,38], blocking both Th2- and Th17-type cytokines [37,39]. IL-27 exerts regulatory functions, mostly by inducing and regulating IL-10 and IL-17 [37,40]. Severe malaria tropica in children and non-healing *Leishmania major* infection in mice were accompanied by depressed levels of IL-27, despite high IL-10 [40,41]. IL-27R-deficient mice were able to control *Toxoplasma gondii* infection initially, but later succumbed due to inflammatory immune responses [42]; these mice will develop severe lung inflammation, elevated IgE levels, and eosinophilia [43]. Still, these regulatory properties of IL-27 cannot be adopted universally, as disruption of the IL-27 signaling pathway did not alter egg-induced immunopathology in an experimental schistosomiasis model [44]. While no differences in IL-27 production were observed following stimulation with parasite antigens between the age groups, enhanced IL-27 in adults and the elderly may reflect the stabilization of a regulatory cytokine network in response to repeated parasite encounter.

Cytokines and chemokines are key players in regulating and polarizing cellular reactivity and antibody responses to pathogens and allergens. Helminth antigens induced Eotaxin-2/CCL24 and MCP-4/CCL13 production in adults and the elderly but not in neonates and children, as similarly observed for IL-19 and IL-33. Eotaxin-2/CCL24 activates Th2-type cytokines and chemoattracts eosinophil and basophil granulocytes. Elevated Eotaxin-2/CCL24 levels have been found in experimental helminth infections [45] and in acutely infected *S. mansoni* patients [12,46], and high Eotaxin-2/CCL24 levels were associated with increased liver damage in *S. mansoni*-infected mice. Following treatment of onchocerciasis patients with ivermectin, Eotaxin-2/CCL24 and MCP-4/CCL13 enhanced suggesting that these chemokines facilitated clearance of *O. volvulus* microfilariae by monocytes and eosinophil granulocytes [47]. Interestingly, protozoan *Entamoeba-*specific antigens depressed both Eotaxin-2/CCL24 and MCP-4/CCL13 in adults and the elderly, but not in children and neonates; such depressed responsiveness might support control *E. histolytica* infection, as elevated levels of Eotaxins were observed in mice with persistent *E. histolytica* infection [48]. The chemokine MCP-4/CCL13 attracts granulocytes, monocytes, and T cells, and it has been proposed as a biomarker in asthma [49] being up-regulated during both Th1- and Th2-type hyper-responses [10]. In the present work, MCP-4/CCL13 and Eotaxin-2/CCL24 were not produced in neonates, but were inducible by helminth antigens in adults and the elderly – an observation pointing to the gradual expansion of the parasite-specific immune response repertoire.

In stark contrast to the above studied cytokines and chemokines, the highest production of MIP-1delta/CCL15 was found in neonates and children, whereas MIP-1delta/CCL15 was low in adults and the elderly (Table 2). Cellular MIP-1delta/CCL15 release in response was not inducible by allergens in any group above baseline levels (Tables 3 and 4). The pro-inflammatory chemokine MIP-1delta/CCL15 attracts neutrophil granulocytes, T cells and monocytes [11]. In the present study MIP-1delta/CCL15 was inducible by *Entamoeba-*specific antigens only. Exposure of human monocytes to live microfilaria of *Brugia malayi* enhanced CCL15 mRNA expression, which was also present in IL-4 induced alternative activated macrophages [50]. With an expiring *O. volvulus* infection, low levels of MIP-1delta/CCL15 were detected in patients [47], while the reduced MIP-1delta/CCL15 observed in adults and the elderly may represent an immune adaptation towards lessened inflammatory responses against *E. histolytica*. Similarly, cellular reactivity towards allergens was highest in neonates and significantly reduced in adults and the elderly.

## Conclusion

In summary, helminth and protozoan antigens distinctly activated in adults Th2-type cytokines, effector cell-attracting chemokines and regulatory components, notably IL-27, while such responsiveness was not yet present in neonates and children. Following repeated exposure to parasites and as a consequence of host inability to prevent or eliminate intestinal helminth or protozoa infections, a repertoire of immune responses evolves with lessened pro-inflammatory and pronounced regulatory cytokines and chemokines – this is required for partial parasite control and also to prevent inadequate and excessive host tissue and organ damage.

## Material and methods

### Population study

The study was conducted at the Centre Hospitalier Regional (CHR) in the Central Region of Togo and approved and authorized by the Togolese Ministry of Health (292/99/MS/CAB, 0407/2007/MMSCAB/DGS, MS/DGS/DRS/RC/No. 220 and No. 261) and by the Ethics Committee of the University Clinics of Tübingen, Germany (No. 188/2008/BO2). A total of 152 individuals were included in the study and grouped by age: neonates (n= 36), children (10-13 yrs, n=35), adults (18-45 yrs, n= 39) and elderly (50-80 yrs, n=42). The children were all attending primary schools in suburban areas of the town of Sokodé (Prefecture Tchaoudjo). Adults were from the village of Bouzalo, near the city of Sokodé. Written consent was obtained from the childrens’ parents prior to participation. Peripheral Blood Mononuclear Cells (PBMC) or else Umbilical Cord Blood Cells (UCBC) was collected from all study participants. Blood, stool and urine samples were collected from children and from adults. Umbilical Cord Blood was obtained from mothers giving birth in the Central Hospital of Sokodé. Written informed consent was obtained from all mothers after thoroughly explaining to them the procedures and risks of this study; to ensure understanding, explanations were given in the local language by the medical stuff during prenatal consultations at the CHR. Pregnant women received antiparasite treatment in line with the national health guidelines of Togo both during prenatal consultations (PC) and after partition. All pregnant women received antimalaria prophylaxis as recommended by national health guidelines - receiving either chloroquine at 300 mg/week, which was taken until partition, or a single dose of sulfadoxine/pyrimethamine at the end of the second trimester of pregnancy as well as a further dose at the beginning of the third. In the 4th month of pregnancy, all women received antihelminth treatment (albendazole, single dose 400 mg) and, after partition, they were treated against intestinal protozoan parasites (metronidazole) in line with the national health guidelines.

### Isolation of peripheral blood mononuclear cells (PBMC) and umbilical cord blood cells (UCBC) and cell culture experiments

Isolation of PBMC was carried out as described earlier [51,52]. In brief, 5-9 ml of venous blood was collected and PBMC were then isolated using Ficoll Density Centrifugation at 340 g for 35 minutes. Cells were collected, washed twice in Roswell Park Memorial Institute (RPMI) media supplemented with 100 U/ml penicillin, 100 μg/ml streptomycin and 0.25 μg/ml amphotericin B (Sigma, St. Louis, MO, USA). Cells were counted and cultured at a concentration of 1×10^6^ cells per well, supplemented in RPMI with 5% heat inactivated Fetal Calf Serum (FCS, Biochrom, Berlin, Germany). Umbilical cord blood was obtained from the placentas of healthy, full-term infants, after the placentas were delivered and separated from same. Blood samples were diluted 1:2 with RPMI (Gibco; Eching, Germany) supplemented with 25 mM HEPES buffer, 100 U/ml penicillin and 100 μg/ml streptomycin, 0.25 μg/ml amphotericin B (as above). Umbilical cord mononuclear blood cells (UCBC) were isolated by Ficoll-Paque density gradient centrifugation at 340 g for 35 min at room temperature. UCBC were collected, washed twice in RPMI (as above) at 1400 rpm for 15 min and adjusted to 1×10^6^/ml in RPMI (as above) supplemented with 10% heat inactivated FCS (as above). Freshly isolated UCBC or PBMC were cultured in 48 well plates in 5% CO_2_ at 37°C and saturated humidity in the presence or absence (baseline) of the following antigens/allergens: *Onchocerca volvulus* antigen (OvAg, final concentration in cell culture 20 μg/ml, ), *Ascaris lumbricoides* antigen (AscAg, final conc. 5 μg/ml), *Entamoeba histolytica* antigen (EhAg, final conc. 10 μg/ml), *Plasmodium falciparum* schizonts (PfAg, final conc. 1×10^8^ schizonts/ml), *Dermatophagoides pteronyssinus* (Dp, final conc. 20 μg/ml), *Dermatophagoides farinae* (Df, final conc. 20 ug/ml), *Aspergillus fumigatus* (Af, final conc. 20 μg/ml), *Candida albicans* (Ca, final conc. 20 μg/ml), for 48 hours at 37°Celsius, 5% CO_2_ and saturated humidity. Cells and cell culture supernatant were then harvested and stored at -20°Celsius for further use.

### Preparation of antigens and allergens

*E. histolytica* antigen (EhAg; trophozoites; strain HM-1 axenic culture) was a gift from Dr. Brigitte Walderich (formerly: Institute for Tropical Medicine, University Clinics of Tübingen, Germany). *A. lumbricoides* or *O. volvulus* adult worms were isolated as described previously [52], then washed in phosphate-buffered saline (PBS) before being transferred into a Ten-Broek tissue grinder and homogenized extensively on ice. The homogenate was then sonicated twice (30% intensity) for 3 min on ice and centrifuged at 16,000 g for 30 min at 4°C. The supernatants were collected and sterile-filtered (0.22 μm), and the protein concentration was then determined by BCA protein assay (Pierce, Rockford, USA). *D. pteronyssinus* (Dp), *D. farinae* (Df), *A. fumigatus* (Af) extracts were all purchased from Allergopharma (Rheinbeck, Germany). Crude antigen extracts of *P. falciparum* schizonts were kindly gifted by Dr. A. Luty and Dr. K. Brustoski (formerly: Institute for Tropical Medicine, University of Tübingen, Germany).

### Parasitological analysis

Analysis for helminth and protozoan infections was carried out as previously described [51,52]. Briefly, fresh stool samples were mixed with saline, dispersed on 2 microscope slides and analyzed for intestinal helminth eggs as well as protozoan cysts and trophozoites. All stool samples were examined using the Kato-Katz technique for quantification of helminth eggs per gram of stool (helm-TEST; Labmaster). *Schistosoma haematobium* eggs were detected by filtration of 10 ml urine (polycarbonate membrane, pore size 12 μm; Whatman). Microfilaria stages of *Mansonella perstans* were detected by microscopic analysis after Ficoll density centrifugation. Malaria Rapid Test (OptiMal™, TCS Biosciences, Birmingham UK) was used to determine infection with *P. falciparum*. Children showing signs of malaria (positive thick blood smears for *Plasmodium spp.* and fever or Malaria Rapid Test-positive) and diarrhea were excluded from the study. None of the children presented with *E. histolytica* trophozoites ingested with red blood cells in stool samples, bloody stools or clinical signs of invasive amoebiasis.

### Cytokine and chemokine determination by enzyme-linked immunosorbent assay (ELISA)

Cell culture supernatants were tested for IL-19, IL-27, IL-33, Eotaxin-2/CCL24, MCP-4/CCL13 and MIP-1 delta/CCL15 using ELISA Assay Kits (R&D Systems). Assays were performed according to guidelines supplied by the manufacturer. Conversion of optical densities (OD) to final concentrations (pg/ml) was calculated by cytokine specific standard curves. Assay detection limits were 30 pg/ml for IL-19, 150 pg/ml for IL-27, 20 pg/ml for IL-33, 8 pg/ml for MCP-4/CCL13, 30 pg/ml for Eotaxin-2/CCL24 and 15 pg/ml for MIP-1 delta/CCL15.

### Statistical data analysis

The statistical package JMP 9.0 (SAS Institute) was used to analyze significant differences between the studied groups. Significant differences in cytokine and chemokine concentrations between studied groups were determined by Analysis of Variance (ANOVA) and Tukey’s Test. Due to multiple comparisons, the level of significance was adjusted by Bonferroni-Holm-method.

## Competing interests

The authors have no competing conflicts of interest to declare.

## Authors’ contributions

CK, MB, JH, AA, CJL, KK, XH: study proposal, data collection, study design, statistical analyses. CK, CJL, PTS, RGG: study design and coordination, interpretation of results, writing of manuscript and revision. CK, MB, AA, CJL, PTS: manuscript drafting and revision. All authors read and approved the final manuscript.

## References

[B1] Supali T Verweij JJ Wiria AE Djuardi Y Hamid F Kaisar MM Wammes LJ van Lieshout L Luty AJ Sartono E Yazdanbakhsh M Polyparasitism and its impact on the immune system Int J Parasitol 2010 40 10 1171 1176 10.1016/j.ijpara.2010.05.003 20580905

[B2] Jombo GT Egah DZ Akosu JT **Intestinal parasitism, potable water availability and methods of sewage disposal in three communities in Benue State,** Nigeria: a survey Ann Afr Med 2007 6 1 17 21 10.4103/1596-3519.55736 18240486

[B3] Grzych JM Pearce E Cheever A Caulada ZA Caspar P Heiny S Lewis F Sher A Egg deposition is the major stimulus for the production of Th2 cytokines in murine *schistosomiasis mansoni* J Immunol 1991 146 4 1322 1327 1825109

[B4] Pearce EJ Caspar P Grzych JM Lewis FA Sher A **Downregulation of Th1 cytokine production accompanies induction of Th2 responses by a parasitic helminth,** Schistosoma mansoni J Exp Med 1991 173 1 159 166 10.1084/jem.173.1.159 1824635 PMC2118762

[B5] Díaz A Allen JE Mapping immune response profiles: the emerging scenario from helminth immunology Eur J Immunol 2007 37 12 3319 3326 10.1002/eji.200737765 18000958

[B6] Guo X Stroup SE Houpt ER Persistence of *Entamoeba histolytica* infection in CBA mice owes to intestinal IL-4 production and inhibition of protective IFN-gamma Mucosal Immunol 2008 1 2 139 146 10.1038/mi.2007.18 19079171

[B7] McCall MB Sauerwein RW Interferon-γ–central mediator of protective immune responses against the pre-erythrocytic and blood stage of malaria J Leukoc Biol 2010 88 6 1131 1143 10.1189/jlb.0310137 20610802

[B8] Köhler C Adegnika AA Van der Linden R Agnandji ST Chai SK Luty AJ Szepfalusi Z Kremsner PG Yazdanbakhsh M Comparison of immunological status of African and European cord blood mononuclear cells Pediatr Res 2008 64 6 631 636 10.1203/PDR.0b013e31818718ba 18679157

[B9] Köhler C Tebo AE Dubois B Deloron P Kremsner PG Luty AJ 1-95/C Study Team Temporal variations in immune responses to conserved regions of *Plasmodium falciparum* merozoite surface proteins related to the severity of a prior malaria episode in Gabonese children Trans R Soc Trop Med Hyg 2003 97 4 455 461 10.1016/S0035-9203(03)90090-3 15259482

[B10] Garcia-Zepeda EA Combadiere C Rothenberg ME Sarafi MN Lavigne F Hamid Q Murphy PM Luster AD Human monocyte chemoattractant protein (MCP)-4 is a novel CC chemokine with activities on monocytes, eosinophils, and basophils induced in allergic and nonallergic inflammation that signals through the CC chemokine receptors (CCR)-2 and -3 J Immunol 1996 157 12 5613 5626 8955214

[B11] Youn BS Zhang SM Lee EK Park DH Broxmeyer HE Murphy PM Locati M Pease JE Kim KK Antol K Kwon BS Molecular cloning of leukotactin-1: a novel human beta-chemokine, a chemoattractant for neutrophils, monocytes, and lymphocytes, and a potent agonist at CC chemokine receptors 1 and 3 J Immunol 1997 159 11 5201 5205 9548457

[B12] Sousa-Pereira SR Teixeira AL Silva LC Souza AL Antunes CM Teixeira MM Lambertucci JR Serum and cerebral spinal fluid levels of chemokines and Th2 cytokines in *Schistosoma mansoni* myeloradiculopathy Parasite Immunol 2006 28 9 473 478 10.1111/j.1365-3024.2006.00896.x 16916371

[B13] Dixon H Blanchard C Deschoolmeester ML Yuill NC Christie JW Rothenberg ME Else KJ The role of Th2 cytokines, chemokines and parasite products in eosinophil recruitment to the gastrointestinal mucosa during helminth infection Eur J Immunol 2006 36 7 1753 1763 10.1002/eji.200535492 16783848

[B14] Hung LY Lewkowich IP Dawson LA Downey J Yang Y Smith DE Herbert DR IL-33 drives biphasic IL-13 production for noncanonical type 2 immunity against hookworms Proc Natl Acad Sci USA 2013 110 1 282 287 10.1073/pnas.1206587110 23248269 PMC3538196

[B15] Yasuda K Muto T Kawagoe T Matsumoto M Sasaki Y Matsushita K Taki Y Futatsugi-Yumikura S Tsutsui H Ishii KJ Yoshimoto T Akira S Nakanishi K Contribution of IL-33-activated type II innate lymphoid cells to pulmonary eosinophilia in intestinal nematode-infected mice Proc Natl Acad Sci USA 2012 109 9 3451 3456 10.1073/pnas.1201042109 22331917 PMC3295287

[B16] Jones LA Roberts F Nickdel MB Brombacher F McKenzie AN Henriquez FL Alexander J Roberts CW IL-33 receptor (T1/ST2) signalling is necessary to prevent the development of encephalitis in mice infected with *Toxoplasma gondii* Eur J Immunol 2010 40 2 426 436 10.1002/eji.200939705 19950183

[B17] Humphreys NE Xu D Hepworth MR Liew FY Grencis RK IL-33, a potent inducer of adaptive immunity to intestinal nematodes J Immunol 2008 180 2443 2449 18250453 10.4049/jimmunol.180.4.2443

[B18] Liao SC Cheng YC Wang YC Wang CW Yang SM Yu CK Shieh CC Cheng KC Lee MF Chiang SR Shieh JM Chang MS IL-19 induced Th2 cytokines and was up-regulated in asthma patients J Immunol 2004 173 6712 6718 15557163 10.4049/jimmunol.173.11.6712

[B19] Hsing CH Chiu CJ Chang LY Hsu CC Chang MS IL-19 is involved in the pathogenesis of endotoxic shock Shock 2008 29 1 7 15 18246602 10.1097/shk.0b013e318067de40

[B20] Hunter CA Kastelein R Interleukin-27: balancing protective and pathological immunity Immunity 2012 37 6 960 969 10.1016/j.immuni.2012.11.003 23244718 PMC3531794

[B21] Findlay EG Greig R Stumhofer JS Hafalla JC de Souza JB Saris CJ Hunter CA Riley EM Couper KN Essential role for IL-27 receptor signaling in prevention of Th1-mediated immunopathology during malaria infection J Immunol 2010 185 4 2482 2492 10.4049/jimmunol.0904019 20631310

[B22] Rosário AP F d Lamb T Spence P Stephens R Lang A Roers A Muller W O’Garra A Langhorne J IL-27 promotes IL-10 production by effector Th1 CD4+ T cells: a critical mechanism for protection from severe immunopathology during malaria infection J Immunol 2012 188 1178 1190 10.4049/jimmunol.1102755 22205023 PMC3272378

[B23] Falcone FH Rossi AG Sharkey R Brown AP Pritchard DI Maizels RM *Ascaris suum*-derived products induce human neutrophil activation via a G protein-coupled receptor that interacts with the interleukin-8 receptor pathway Infect Immun 2001 69 6 4007 4018 10.1128/IAI.69.6.4007-4018.2001 11349070 PMC98463

[B24] Salata RA Ahmed P Ravdin JI Chemoattractant activity of *Entamoeba histolytica* for human polymorphonuclear neutrophils J Parasitol 1989 75 4 644 646 10.2307/3282920 2547924

[B25] Wolk K Kunz S Asadullah K Sabat R Cutting edge: immune cells as sources and targets of the IL-10 family members? J Immunol 2002 168 11 5397 5402 12023331 10.4049/jimmunol.168.11.5397

[B26] Zhong H Wu Y Belardinelli L Zeng D A2B adenosine receptors induce IL-19 from bronchial epithelial cells, resulting in TNF-alpha increase Am J Respir Cell Mol Biol 2006 35 5 587 592 10.1165/rcmb.2005-0476OC 16778150

[B27] Hofmann SR Rösen-Wolff A Tsokos GC Hedrich CM Biological properties and regulation of IL-10 related cytokines and their contribution to autoimmune disease and tissue injury Clin Immunol 2012 143 2 116 127 10.1016/j.clim.2012.02.005 22459704

[B28] Azuma YT Matsuo Y Kuwamura M Yancopoulos GD Valenzuela DM Murphy AJ Nakajima H Karow M Takeuchi T Interleukin-19 protects mice from innate-mediated colonic inflammation Inflamm Bowel Dis 2010 16 6 1017 1028 10.1002/ibd.21151 19834971

[B29] Gallagher G Eskdale J Jordan W Peat J Campbell J Boniotto M Lennon GP Dickensheets H Donnelly RP Human interleukin-19 and its receptor: a potential role in the induction of Th2 responses Int Immunopharmacol 2004 4 5 615 626 10.1016/j.intimp.2004.01.005 15120647

[B30] Schmitz J Owyang A Oldham E Song Y Murphy E McClanahan TK Zurawski G Moshrefi M Qin J Li X Gorman DM Bazan JF Kastelein RA IL-33, an interleukin-1-like cytokine that signals via the IL-1 receptor-related protein ST2 and induces T helper type 2-associated cytokines Immunity 2005 23 5 479 490 10.1016/j.immuni.2005.09.015 16286016

[B31] Liew FY IL-33: a Janus cytokine Ann Rheum Dis 2012 71 Suppl 2 i101 i104 10.1136/annrheumdis-2011-200589 22460136

[B32] Kamekura R Kojima T Takano K Go M Sawada N Himi T The role of IL-33 and its receptor ST2 in human nasal epithelium with allergic rhinitis Clin Exp Allergy 2012 42 2 218 228 10.1111/j.1365-2222.2011.03867.x 22233535

[B33] Moussion C Ortega N Girard JP The IL-1-like cytokine IL-33 is constitutively expressed in the nucleus of endothelial cells and epithelial cells in vivo: a novel ‘alarmin’? PLoS One 2008 3 10 e3331 10.1371/journal.pone.0003331 18836528 PMC2556082

[B34] Maizels RM Hewitson JP Smith KA Susceptibility and immunity to helminth parasites Curr Opin Immunol 2012 24 4 459 466 10.1016/j.coi.2012.06.003 22795966 PMC3437973

[B35] Hepworth MR Danilowicz-Luebert E Rausch S Metz M Klotz C Maurer M Hartmann S Mast cells orchestrate type 2 immunity to helminths through regulation of tissue-derived cytokines Proc Natl Acad Sci USA 2012 109 17 6644 6649 10.1073/pnas.1112268109 22493240 PMC3340035

[B36] Wills-Karp M Rani R Dienger K Lewkowich I Fox JG Perkins C Lewis L Finkelman FD Smith DE Bryce PJ Kurt-Jones EA Wang TC Sivaprasad U Hershey GK Herbert DR Trefoil factor 2 rapidly induces interleukin 33 to promote type 2 immunity during allergic asthma and hookworm infection J Exp Med 2012 209 3 607 622 10.1084/jem.20110079 22329990 PMC3302229

[B37] Yoshida H Miyazaki Y Regulation of immune responses by interleukin-27 Immunol Rev 2008 226 234 247 10.1111/j.1600-065X.2008.00710.x 19161428

[B38] Villarino AV Huang E Hunter CA Understanding the pro- and anti-inflammatory properties of IL-27 J Immunol 2004 173 2 715 720 15240655 10.4049/jimmunol.173.2.715

[B39] Sturmhofer JS Hunter CA Advances in understanding the anti-inflammatory properties of IL-27 Immunol Lett 2008 117 2 123 130 10.1016/j.imlet.2008.01.011 18378322 PMC2390685

[B40] Anderson CF Sturmhofer JS Hunter CA Sacks D IL-27 regulates IL-10 and IL-17 from CD4+ cells in non-healing *Leishmania major* infection J Immunol 2009 183 7 4619 4627 10.4049/jimmunol.0804024 19748991 PMC2749572

[B41] Ayimba E Hegewald J Ségbéna AY Gantin RG Lechner CJ Agosssou A Banla M Soboslay PT Proinflammatory and regulatory cytokines and chemokines in infants with uncomplicated and severe *Plasmodium falciparum* malaria Clin Exp Immunol 2011 166 2 218 226 10.1111/j.1365-2249.2011.04474.x 21985368 PMC3219897

[B42] Villarino A Hibbert L Lieberman L Wilson E Mak T Yoshida H Kastelein RA Saris C Hunter CA The IL-27R (WSX-1) is required to suppress T cell hyperactivity during infection Immunity 2003 19 5 645 655 10.1016/S1074-7613(03)00300-5 14614852

[B43] Miyazaki Y Inoue H Matsumura M Matsumoto K Nakano T Tsuda M Hamano S Yoshimura A Yoshida H Exacerbation of experimental allergic asthma by augmented Th2 responses in WSX-1-deficient mice J Immunol 2005 175 2401 2407 16081811 10.4049/jimmunol.175.4.2401

[B44] Shainheit MG Saraceno R Bazzone LE Rutitzky LI Stadecker MJ Disruption of interleukin-27 signaling results in impaired gamma interferon production but does not significantly affect immunopathology in murine schistosome infection Infect Immun 2007 75 6 3169 3177 10.1128/IAI.01053-06 17403877 PMC1932859

[B45] Perry CR Burke ML Stenzel DJ McManus DP Ramm GA Gobert GN Differential expression of chemokine and matrix re-modelling genes is associated with contrasting schistosome-induced hepatopathology in murine models PLoS Negl Trop Dis 2011 5 6 e1178 10.1371/journal.pntd.0001178 21666794 PMC3110159

[B46] Silveira-Lemos D Teixeira-Carvalho A Martins-Filho OA Souza-Soares AL Castro-Silva P Costa-Silva MF Guimarães PH Ferraz HB Oliveira-Fraga LA Teixeira MM Corrêa-Oliveira R Seric chemokines and chemokine receptors in eosinophils during acute human *schistosomiasis mansoni* Mem Inst Oswaldo Cruz 2010 105 4 380 386 10.1590/S0074-02762010000400006 20721479

[B47] Lechner CJ Gantin RG Seeger T Sarnecka A Portillo J Schulz-Key H Karabou PK Helling-Giese G Heuschkel C Banla M Soboslay PT Chemokines and cytokines in patients with an occult *Onchocerca volvulus* infection Microbes Infect 2012 14 5 438 446 10.1016/j.micinf.2011.12.002 22202179

[B48] Rojas-López AE Soldevila G Meza-Pérez S Dupont G Ostoa-Saloma P Wurbel MA Ventura-Juárez J Flores-Romo L García-Zepeda EA CCR9+ T cells contribute to the resolution of the inflammatory response in a mouse model of intestinal amoebiasis Immunobiology 2012 217 8 795 807 10.1016/j.imbio.2012.04.005 22633147

[B49] Kalayci O Sonna LA Woodruff PG Camargo CA Luster AD Lilly CM Monocyte chemotactic protein-4 (MCP-4; CCL-13): a biomarker of asthma J Asthma 2004 41 1 27 33 10.1081/JAS-120024590 15046375

[B50] Semnani RT Mahapatra L Moore V Sanprasert V Nutman TB Functional and phenotypic characteristics of alternative activation induced in human monocytes by interleukin-4 or the parasitic nematode *Brugia malayi* Infect Immun 2011 79 10 3957 3965 10.1128/IAI.05191-11 21788379 PMC3187236

[B51] Hamm DM Agossou A Gantin RG Kocherscheidt L Banla M Dietz K Soboslay PT Coinfections with *Schistosoma haematobium*, *Necator americanus*, and *Entamoeba histolytica/Entamoeba dispa*r in children: chemokine and cytokine responses and changes after antiparasite treatment J Infect Dis 2009 199 11 1583 1591 10.1086/598950 19392635

[B52] Soboslay PT Hamm DM Pfäfflin F Fendt J Banla M Schulz-Key H Cytokine and chemokine responses in patients co-infected with *Entamoeba histolytica*/dispar, *Necator americanus* and *Mansonella perstans* and changes after anti-parasite treatment Microbes Infect 2006 8 1 238 247 10.1016/j.micinf.2005.06.019 16239120

